# Supplementary material for: Circulating Serum VEGF, IGF-1 and MMP-9 and Expression of Their Genes as Potential Prognostic Markers of Recovery in Post-Stroke Rehabilitation—A Prospective Observational Study
Source: Brain Sci. 2023 May 23;13(6):846. doi: 10.3390/brainsci13060846 (PMC10296181; doi:10.3390/brainsci13060846)
Supplement: Supplementary file 1 [file brainsci-13-00846-s001.zip › brainsci-2354123-supplementary.pdf]

Supplementary Material. Comparison of parametric and non-parametric test results

Results presented in Table 2:

Variable	*P*-value		Is statistical inference the same?
	Parametric test presented in the manuscript body	Respective non-parametric test [a]	
ADL	0.0008	<0.0001	Yes
Rankin	0.0024	0.0002	Yes
NIHSS	0.0059	0.0001	Yes
MOCA/MMSE	0.0004	0.0008	Yes
BDI/GDS	0.0162	0.0004	Yes

[a] Wilcoxon signed-rank test

Results presented in Figure 1:

Variable	*P*-value		Is statistical inference the same?
	Parametric test presented in the manuscript body	Respective non-parametric test [a]	
MMP9 protein	0.0032	0.0075	Yes
VEGF protein	0.0112	0.0083	Yes
IGF1 protein	0.1730	0.1843	Yes
MMP9 mRNA	0.2801	0.1347	Yes
VEGF-A mRNA	0.3780	0.0961	Yes

[a] Wilcoxon signed-rank test

Results presented in Table 3:

Variable	*P*-value		Is statistical inference the same?
	Parametric test presented in the manuscript body	Respective non-parametric test [a]	
Cognitive improvement			
Δ MMP9 protein	0.0034	0,0019	Yes
Δ VEGF protein	0.9333	0,9011	Yes
Δ IGF1 protein	0.8721	0,7905	Yes
Δ *MMP9* mRNA	0.8974	0,1783	Yes
Δ *VEGF-A* mRNA	0.2257	0,9704	Yes
Improvement in depression			
Δ MMP9 protein	0.8878	0.6876	Yes
Δ VEGF protein	0.0427	0.0601	No
Δ IGF1 protein	0.7083	0.3767	Yes
Δ *MMP9* mRNA	0.1076	0.9442	Yes
Δ *VEGF-A* mRNA	0.0117	0.1928	No

[a] Spearman’s rho

Results presented in Table 4:

Variable	*P*-value		Is statistical inference the same?
	Parametric test presented in the manuscript body	Respective non-parametric test [a]	
Cognitive improvement			
pre-reh MMP9 protein	0.1402	0.2803	Yes
pre-reh VEGF protein	0.8603	0.3382	Yes
pre-reh IGF1 protein	0.6575	0.3119	Yes
pre-reh *MMP9* mRNA	0.0127	0.0014	Yes
pre-reh *VEGF-A* mRNA	0.4084	0.0495	No
Improvement in depression			
pre-reh MMP9 protein	0.3504	0.9157	Yes
pre-reh VEGF protein	0.0218	0.1076	No
pre-reh IGF1 protein	0.5225	0.9490	Yes
pre-reh *MMP9* mRNA	0.4319	0.1626	Yes
pre-reh *VEGF-A* mRNA	0.1499	0.5673	Yes

Pre-reh – pre-rehabilitation

[a] Spearman's rho
